# Two-Photon Uncaging of Glutamate

**DOI:** 10.3389/fnsyn.2018.00048

**Published:** 2019-01-09

**Authors:** Graham C. R. Ellis-Davies

**Affiliations:** Department of Neuroscience, Mount Sinai School of Medicine, New York, NY, United States

**Keywords:** Glu = glutamate, 2-photon, uncaging, quanta, plasticity, dendritic spikes

## Abstract

Two-photon microscopy produces the excited singlet state of a chromophore with wavelengths approximately double that used for normal excitation. Two photons are absorbed almost simultaneously, via a virtual state, and this makes the excitation technique inherently non-linear. It requires ultra-fast lasers to deliver the high flux density needed to access intrinsically very short lived intermediates, and in combination with lenses of high numerical aperture, this confines axial excitation highly. Since the two-photon excitation volume is similar to a large spine head, the technique has been widely used to study glutamatergic transmission in brain slices. Here I describe the principles of two-photon uncaging of glutamate and provide a practical guide to its application.

Caged compounds are, by definition, biological molecules which have been rendered inert by covalent attachment of a photochemical protecting group (Ellis-Davies, 2000). Originating in 1978 with caged ATP (Kaplan et al., 1978), all important biological signaling molecules and cations have been controlled by uncaging (Ellis-Davies, 2007). Conceptually simple in its design, the strategy is to block a crucial functionality of the biomolecule that is required for its activity with the “caging chromophore.” Irradiation cuts this bond, releasing the caged substrate. The term “caged compounds” was coined by a physiologist (Joe Hoffman) who was unaware of the term “caged” was used in chemistry to mean box-like structures. And we should not forget, of course, those involved in animal husbandry use the term in a literal way. The simplicity of term remains attractive, so I use it in the way Hoffman did, to mean a *functional* cage.

After the initial success of caged ATP in the study of the Na,K-ATPase (Kaplan and Hollis, 1980), other biologists became interested in caged compounds. Henry Lester, George Hess, David Trentham and Roger Tsien and their co-workers all made seminal contributions to the field with the development of caged cGMP (Lester et al., 1979), carbamoylcholine (Walker et al., 1986), IP_3_ (Walker et al., 1987), and Ca^2+^ (Tsien and Zucker, 1986) in the 1980–86 period. All these caged compounds were designed for photolysis with near-UV light using the *ortho*-nitrobenzyl photochemical protecting group introduced by Barltrop et al. (1966). As an interesting aside I would like to point out that Barltrop was a natural product chemist who did a sabbatical with Melvin Calvin in the 1950s, with whom he must have started to think about using light for synthetic organic chemistry, and that Barltrop's work eventually lead to the gene chip revolution (McGall et al., 1997). For neuroscience, the work of the Hess group was crucial, as they developed the first caged neurotransmitters (Wilcox et al., 1990; Wieboldt et al., 1994a,b; Niu et al., 1996; Breitinger et al., 2000). And so laser uncaging of neurotransmitters became a topic of active research for neurophysiologists using one-photon (1P) photolysis (Eder et al., 2004). Beyond low-resolution functional mapping of receptors (Eder et al., 2004), 1P uncaging has been widely used for studying circuit connectivity by many laboratories (Shepherd, 2012). Such studies will not be discussed here.

The challenge for using 1P uncaging of glutamate for high-resolution functional mapping is that normal excitation must release the neurotransmitter wherever light hits the solution of caged compound. Of course lenses with a high numerical aperture will produce focused light, thus glutamate concentrations will be maximal at the focal point. But the same quantity of glutamate will be released in every plane above and below this point because of linear excitation. Thus, in complex biological preparations, such as brain slices this can lead to large clouds of glutamate release outside the site of interest. One can immediately appreciate that 2P uncaging is potentially very advantageous for glutamate neurophysiology, as uncaging becomes pin-point due the nature of non-linear excitation (Figure 1A).

**Figure 1:**
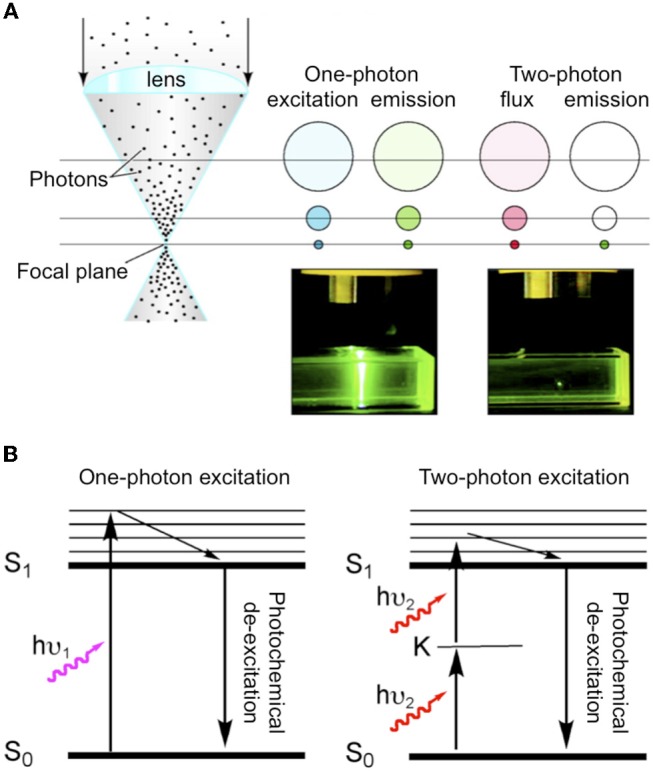
Comparison of one-and two-photon excitation. **(A)** Cartoon illustrating the fundamental differences between normal (i.e., one-photon) absorption and two-photon absorption. As blue light is focused by a lens during one-photon excitation of a chromophore the concentration of excited molecules increases but the same number of molecules are excited in each z section. Whereas, during two-photon irradiation with NIR the total flux in each z section is constant (red), but excitation only occurs at the focus (green). Hence, for fluorescence emission can be seen throughout the light path for one-photon, but is pin-point for two-photon. Adapted from Ellis-Davies (2011b), by permission of the American Chemical Society. **(B)** Simple Jablonski diagram illustrating the absorption of light from the ground state (S_0_) to excited state (S_1_) occurs directly with one-photon absorption (hu_1_), and goes via a “virtual state” K in the case of two-photon excitation (hu_2_).

## A Short History of 2-photon Excitation

Quantum theory says that absorption of light occurs when the transition moment between the ground state and the excited state is non-zero. This is only true when there is an inversion of orbital symmetry during the transition (Electronic states have symmetries that are either even, *gerade* or “*g*” states, or uneven, *ungerade*, or “*u*” states). This principle is formalized in the parity selection rule for light absorption: transitions from *g* to u or from *u* to *g* are allowed; transitions from *g* to *g*, or from *u* to *u* are forbidden. Two-photon excitation gives rise to “virtual” states in which the electronic wave function remains unchanged when the Cartesian coordinate system of the molecule is inverted through the center of symmetry, and so this process is strictly “geometrically forbidden” by the parity selection rule. Göppert-Mayer (1931) realized that Paul Dirac's dispersion theory could apply to 2P *excitation* as well as light transmission. She developed the idea of an “intermediate electronic excited state” (K in Figure 1B) that must have opposite symmetry of the ground and final excited state (so the parity rule for light absorption still applies), but overall the selection rule for 2P transitions is the exact opposite of 1P: *g* to *g* is allowed but *g* to *u* is forbidden (Friedrich, 1982).

The development of lasers revolutionized molecular spectroscopy, and in the 1960s and 70s 2P excitation was used to study electronic excitations that had only been known theoretically [i.e., the forbidden *g* to *g* transitions, and especially *gerade* excited state energy levels (Friedrich, 1982)]. Birge et al were the first to use 2P excitation to study biological chromophores, such as rhodopsin (Birge, 1986). In 1978 Sheppard and Kampfner suggested 2P excitation might be used for non-linear scanning microscopy (Sheppard and Kampfner, 1978). But it was not until the pioneering work of Denk et al. (1990) that this idea was realized (Denk et al., 1990). Of course since that time 2P imaging has become a standard optical method (Denk and Svoboda, 1997; Soeller and Cannell, 1999; Zipfel et al., 2003; Helmchen and Denk, 2005; Ellis-Davies, 2011b; Crowe and Ellis-Davies, 2014). In the abstract of their seminal study Denk also observed that: “This technique also provides unprecedented capabilities for three-dimensional, spatially resolved photochemistry, particularly photolytic release of caged effector molecules” (Denk et al., 1990).

**Figure 2:**
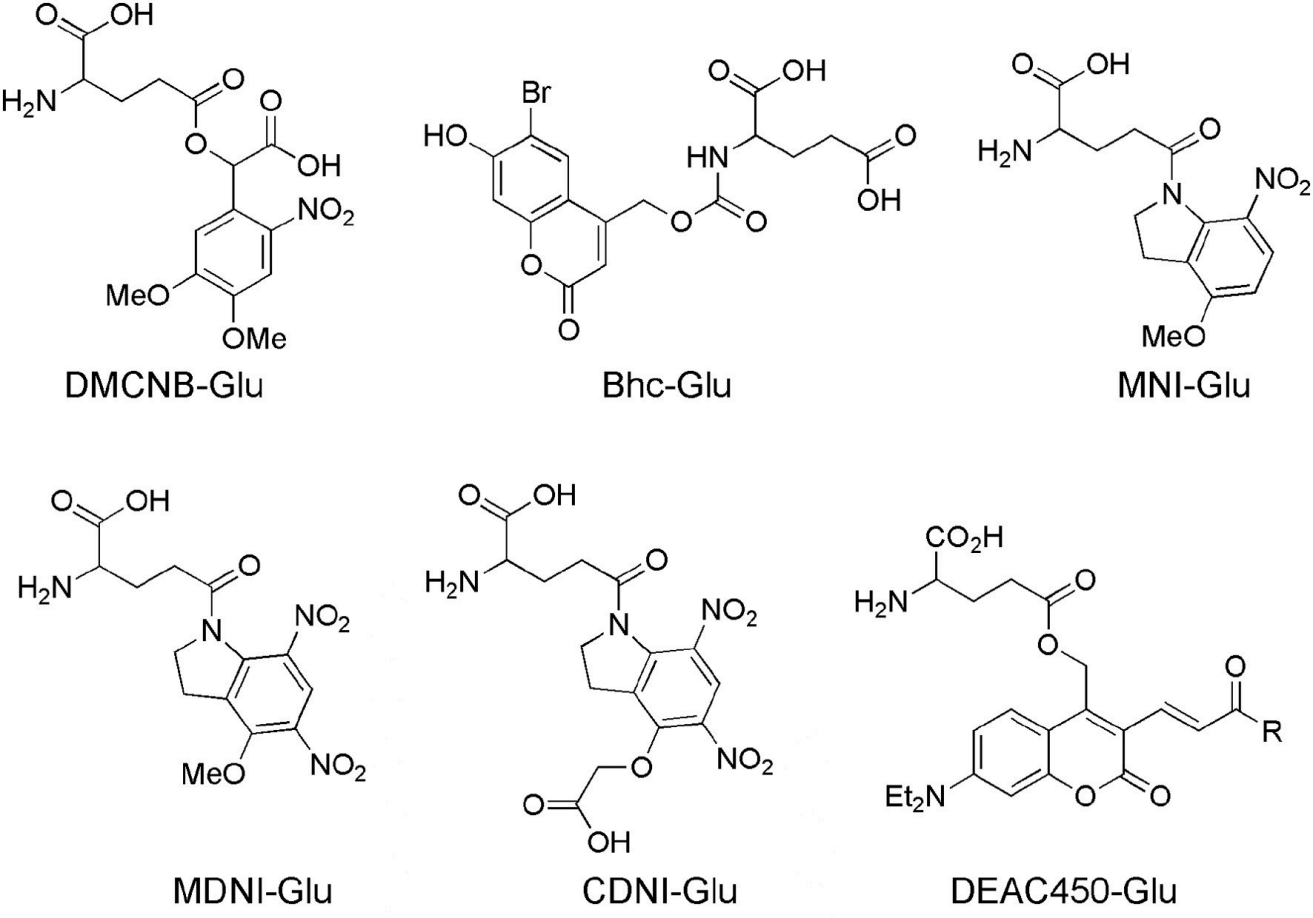
Caged glutamate probes designed for 2P photolysis. Structures of caged glutamate probes used for 2P photolysis. DMCNB, (Ellis-Davies, 1999), Bhc, (Furuta et al., 1999); MNI, (Matsuzaki et al., 2001); MDNI, (Fedoryak et al., 2005); CDNI, (Ellis-Davies et al., 2007a); DEAC450, (Olson et al., 2013b).

In fact, uncaging was starting to mature as a technique by 1990, however uncaging of neurotransmitters was essentially nascent at that point, so Denk's observation proved to be extremely prescient. Glutamate was not uncaged in brain slices until 1993 (Callaway and Katz, 1993). Denk himself published the first proof of principle 2P uncaging experiment a year later (Denk, 1994). Until 1999 no further reports of 2P photolysis in living cells appeared. Lipp and Niggli uncaged Ca^2+^ by irradiation of DM-nitrophen (Kaplan and Ellis-Davies, 1988) to mimic Ca^2+^ sparks to initiate Ca^2+^ waves in cardiac myocytes (Lipp and Niggli, 1998). Their work suggested to me that the electron donating groups of the DM-nitrophen chromophore conferred sufficient absorptivity upon of the *ortho*-nitrobenzyl chromophore to make it reasonably sensitive to 2P excitation and useful for highly localized uncaging. This discovery lead to the synthesis of DMNPE-4 (Ellis-Davies, 1998) and DMCNB-glutamate (Figure 2), both of which are 2P sensitive (DelPrincipe et al., 1999; Ellis-Davies, 1999).

Starting in 1999 several new caged glutamate probes were developed by organic chemists. I give brief survey of these, starting with Roger Tsien's contribution to this field, which was his inaugural paper to *PNAS* on his election to US National Academy of Sciences.

### Bhc-Glu

The bromo-hydroxycoumarin (Bhc) probe that was specifically designed for 2P photolysis of glutamate (Furuta et al., 1999). Based around the coumarin photochemical protecting group which had first been used for uncaging cAMP (Furuta et al., 1995) with near-UV, Bhc-Glu (Figure 2) has a 2P cross-section of 50 GM, and a quantum yield of photolysis of 0.019, giving a probe with photochemical properties of great potential. However, actually caged via the carbamate, the known (Corrie et al., 1993) slow rate of hydrolysis of the photoproduct (which is *not* glutamate) meant Bhc-Glu eventually released the neurotransmitter on a slow time scale of 10 ms. The residence time of excited molecules on the 2P focal volume is 0.3 ms (Brown et al., 1999). Furthermore, the chromophore itself is quite lipophilic causing severe solubility issues for practical use in brain slices. Never the less, Bhc-Glu was important for the field as it pointed the way for future developments, and highlighted the difficulties in making an “ideal cage” for neurophysiology.

### MNI-Glu

**Figure 3:**
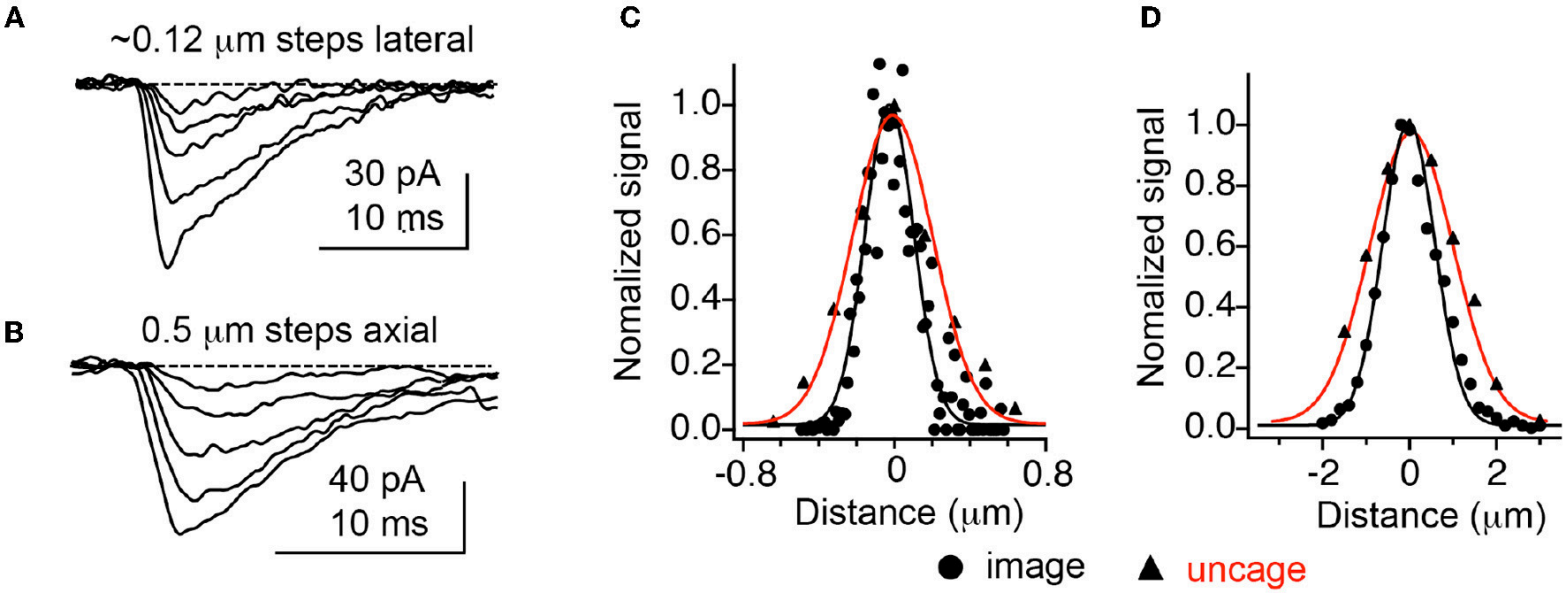
Optical resolution compared with 2P uncaging currents at single spines. **(A,B)** Currents decrease at an isolated spine on a CA1 neuron as the uncaging laser is moved away from the cell laterally and axially. **(C,D)** Comparison of currents evoked by 2P uncaging of MNI-Glu (red curves) with diffraction-limited 2P imaging of 0.1 mm fluorescent beads. Adapted from Smith et al. (2003), by permission of the Physiological Society.

The first caged glutamate that worked well for 2P uncaging was 4-methoxy-7-nitroindolinyl-glutamate (MNI-Glu, Figure 2). Independently synthesized by myself (Matsuzaki et al., 2000) and the Corrie laboratory in 2000 (Papageorgiou and Corrie, 2000). Built on knowledge gained from DM-nitrophen and DMCNB-Glu (Ellis-Davies, 1999), that electron-rich nitroaromatic chromophores were able to undergo efficacious 2PE, I reasoned that adding such substituents to nitroindolines would probably allow effective 2P uncaging in brain slices. Such proved to be the case. In 2001 my collaboration with Kasai and co-workers set the foundations for the use of 2P uncaging of MNI-Glu (Matsuzaki et al., 2001) by many other neurophysiologists (see below). MNI-Glu has proved useful as it possesses a unique set of properties in terms of a caged Glu probe: (1) It is biologically inert toward AMPA-R. Remarkably, even at 12 mM there is no antagonism apparent. It was also reported initially that MNI-Glu was inert toward GABA-A receptors (Canepari et al., 2001), but this proved not to be the case subsequently. (2) It is highly stable at physiological pH. Solutions used for 1 day at 25–37°C also show no hydrolysis, and stored at 4°C for 4 days also show no hydrolysis (Huang et al., 2005). (3) It is highly soluble in physiological buffer, solutions of at least 200 mM can be made. (4) It is photolyzed with good efficiency by near-UV light, with a quantum yield in the 0.065–0.085 range (Papageorgiou and Corrie, 2000; Corrie et al., 2016). (5) It absorbs light well in the near-UV (extinction coefficient 4,500 mM cm at 336 nm), and can be photolyzed with violet lasers that are standard on confocal microscopes as the absorption in about 10% of the maximum. (6) It has a 2P uncaging cross-section of 0.06 GM at 730 nm (Matsuzaki et al., 2001), a value sufficient to allow many experiments without apparent phototoxicity (see below). (7) These absorptions make MNI-Glu optical compatible with other chromophores used for fluorescence imaging, such as GFP, YFP, most Ca^2+^ dyes, and dyes, such as Alexa-594. (8) Glutamate is released quickly, as judged by the rapid rise times of photo-evoked currents produced by 50 ms flashes (Matsuzaki et al., 2001). (9) 2P uncaging at single spines shows excellent 3D resolution (Figure 3) (10) MNI-Glu can be made easily in five steps from readily available starting material. After the initial success of MNI-Glu for 2P uncaging experiments in brain slices in 2001 (Matsuzaki et al., 2001), the probe became commercially available from Tocris in about 2003. It should be noted that we used the trifluoroacetic acid (TFA) salt in 2001 (Matsuzaki et al., 2001), and zwitterionic MNI-Glu (i.e., desalted, non-TFA compound) in 2003 (Smith et al., 2003), and it was the latter that was commercialized by Tocris. Of course, once in solution the TFA counter ion freely dissociates from the caged compound as it is dissolved in physiological buffer.

With MNI-Glu having such a nice combination of chemical, photochemical and pharmacological properties, what scope for probe development remained? Three properties were open to chemical ingenuity: (a) increase in quantum yield, allowing less light for photolysis, potentially enabling longer term experiments; (b) improvements in GABA-A receptor pharmacology; and (c) photolysis at longer wavelengths, allowing 2-color uncaging of two biomolecules with chromatic independence.

### MDNI-Glu

4-Methoxy-5,7-dinitroindolinyl-glutamate (MDNI-Glu) was the probe that first attempted to improve the quantum yield of uncaging (Fedoryak et al., 2005). We found that this probe was photolyzed with a quantum yield of about 0.5. We also found that in a cuvette 2P photolysis of MDNI-Glu was about 5–6 times more effective than MNI-Glu suggesting the second nitro group merely increases the quantum yield. Uncaging MDNI-Glu with a violet laser enabled extremely efficacious photo-evoked Ca^2+^ signals in astrocytes in brain slices (Fedoryak et al., 2005). Recently our probe has been remade in Hungary, with the claim that it was a “novel invention,” with exactly same molecule was called “DNI-Glu” (Chiovini et al., 2014).

### CDNI-Glu

We found there were some practical issues of solubility with MDNI-Glu, so addressed these by adding one carboxylate to methoxy group. Thus, 4-carboxymethoxy-5,7-dinitroindolinyl-glutamate (CDNI-Glu) was introduced in 2007. This probe maintained (Ellis-Davies et al., 2007a) the chemical properties of MDNI-Glu. In collaboration with the groups of Bergles and Kasai we showed it performed well on neurons photochemically. The Bergles group tested CDNI-Glu against MNI-Glu “blind,” with solutions of both probes bath-applied to brain slices in succession to same neuron multiple times, the dramatic difference in current responses was striking. The Kasai group tested the same compounds by local perfusion of each probe to neurons in brain slices, 2P uncaging revealed that CDNI-Glu was about five times larger than MNI-Glu. The Bergles group saw similar results in their 2P experiments (Ellis-Davies, 2011b). Interestingly, conditions for 2P uncaging could be found where multiple uncaging events on single spines with MNI-Glu were phototoxic, but not for CDNI-Glu as less energy was required to evoke the same current in the latter case (Ellis-Davies et al., 2007a).

### CDNI-GABA

In 2010 we introduced the CDNI-caged version of GABA (Matsuzaki et al., 2010). This was the first compound used for efficient and effective 2P uncaging of GABA on neurons in brain slices. The quantum yield of uncaging of photolysis is slightly higher than CDNI-Glu, being 0.6. It is useful to note that both compounds were made with no silica gel chromatography being required. However, the last synthetic step, addition of the crucial second nitro group, requires quite harsh conditions, so the final reaction mixture requires HPLC purification with TFA, leading to the isolation of the TFA salt of the CDNI cage (Ellis-Davies, 2011a). While a detailed discussion of caged GABA is beyond the scope of a review on 2P uncaging of glutamate, I would note that CDNI-GABA has proved quite useful in several reports (Chiu et al., 2013; Gross et al., 2013; Oh et al., 2016; Villa et al., 2016). In particular, in a very elegant study by Kwon and co-workers on synaptogenesis during development induced by GABA (Oh et al., 2016).

### DEAC450-Glu

**Figure 4:**
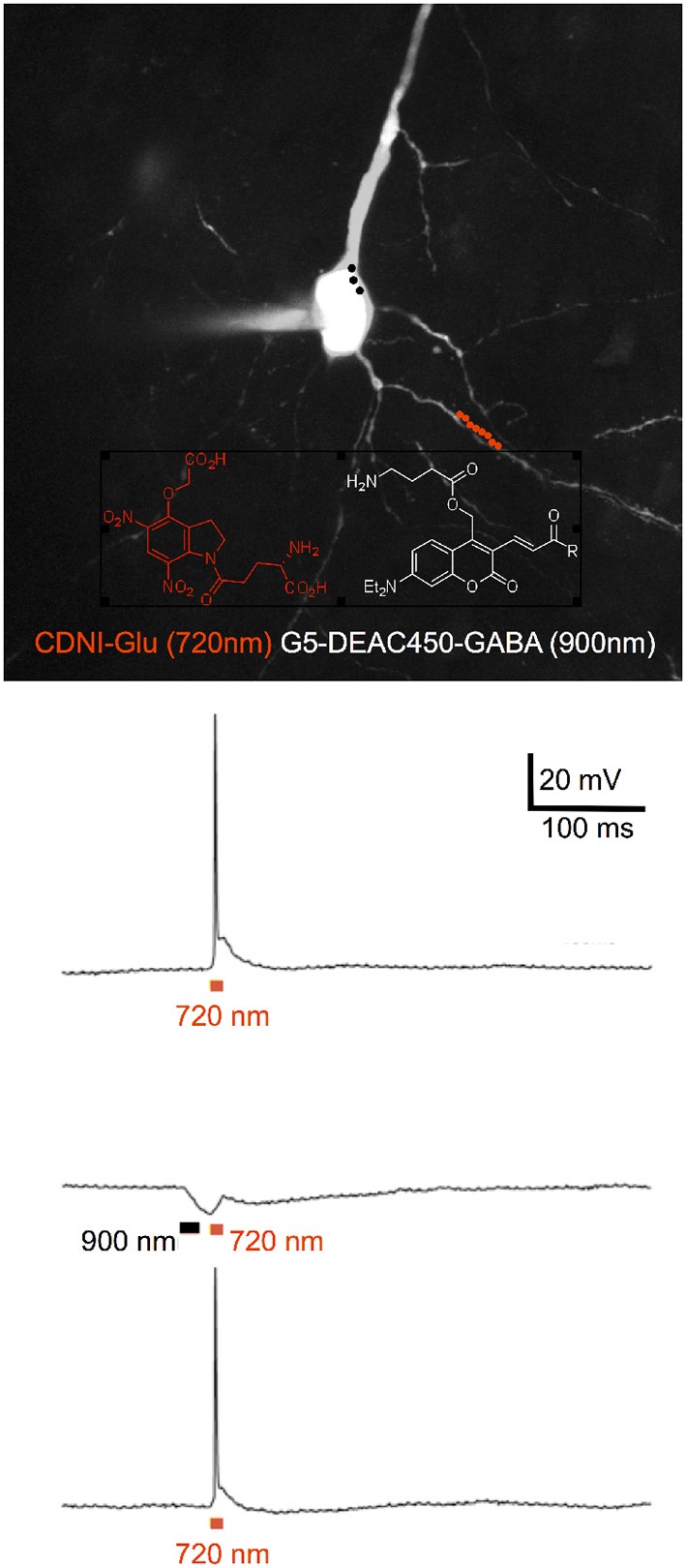
Two-color 2P uncaging of glutamate and GABA. CDNI-Glu (1 mM) and G5-DEAC450-GABA (0.6 mM) were bath applied to a brain slice. The red and black dots indicate the location for irradiation at 720 nm along a dendrite (red) and 900 nm around the soma (black). Uncaging at 720 nm (10 × 1 ms, 50 mW) fired an action potential (top trace), which could be blocked (middle trace) by prior uncaging at 900 nm (3 × 3 ms, 50 mW). Such blockade was found to be reversible (lower trace). Adapted from Richers et al. (2017), by permission from Wiley-VCH.

Simple nitroaromatic caged compounds, such as DM-nitrophen and CDNI-Glu are best photolyzed with a Ti:sapphire laser at red wavelengths around 720–740 nm. They are much less sensitive to photolysis at longer wavelengths, such as 800–830 nm range (Kantevari et al., 2010, Figure 4), imply that at 900 nm these probes are photostable. This longer wavelength “optical window” provides an opening for uncaging biomolecules with second, complementary wavelength of 2P light. Thus, we synthesized a 7-diethylaminocoumarin (DEAC) derivative that absorbs visible light maximally around 450 nm, and is uncaged by 2P excitation best at double this wavelength (Olson et al., 2013b). Crucially the near-UV absorption minimum around 350 nm leads to a very low 2P absorption at 720 nm, the region that is ideal for simple nitroaromatic caged compounds. Thus, when DEAC450-Glu is partnered with CDNI-GABA, or CDNI-Glu with DEAC450-GABA, two-color uncaging experiments with 720 nm and 900 nm are possible (Amatrudo et al., 2015). An example of the latter is shown in Figure 4. In this experiment each compound was bath applied at similar concentrations, irradiation with 720 and 900 nm light independent in current clamp were used to fire/block action potentially with excellent chromatic selectivity using dual 2P uncaging. I would note that DEAC450 is fluorescent in the green range, so is more difficult to use with GFP than MNI, CDNI or RuBi. We have found that normal red dyes (e.g., Alexa-594) are excited in the NIR (Figure 4 uses 1,070 nm for imaging).

Like MNI-Glu and CDNI-Glu, DEAC450-Glu, and DEAC450-GABA antagonize GABA-A receptors (Olson et al., 2013b; Amatrudo et al., 2014). Indeed all caged Glu and GABA probes we have tested are antagonistic. Interestingly, the rutheniumbipyridials RuBi-Glu and RuBi-GABA have very similar IC-50 values (7.8 and 4.4 mM, Table 1), providing clue to the origin of the antagonism, namely that a carboxylate of each probe can enter the GABA binding cleft and act as a competitive antagonist to GABA. The amino acid carboxylate of Glu is probably too close to RuBi to influence binding much, whereas for DEAC450 it has more influence (Table 1). These data suggested that to reduce antagonism dramatically one probably needed to “cover” both ends of the amino acids, thus we developed the concept of “cloaked caged compounds” to deliver biologically inert caged neurotransmitters (Richers et al., 2017).

**Table 1:** GABA-A receptor antagonism of caged neurotransmitters.

**Caged NT**	**IC_**so**_ (μM)**
MNI-Glu	105
CDNI-GABA	110
CDNI-Glu	243
DEAC450-Glu	33
DEAC450-GABA	0.5
PEG-DEAC450-GABA	11
RuBi-Glu	7.7
RuBi-GABA	4.4
G5-DEAC450-GABA	900

*All IC-50s were measured by bath application of the probe to brain slices acutely isolated from adult mice. L2/3 neurons in the PFC were patch-clamped and a stimulation pipette was used to evoke GABAergic input in the presence of AMPA-and NMDA-receptor blockers (Olson et al., 2013b; Amatrudo et al., 2014; Richers et al., 2017)*.

### Cloaked Caged Neurotransmitters

The initial pharmacological characterization of MNI-Glu by Corrie and Ogden in 2001 suggested that this probe was inert toward both AMPA and GABA-A receptors (Canepari et al., 2001). We did not evaluate inhibitory pharmacology for the simple reason that we did not expect any off-target issues for caged Glu. Around the same time, the first report of caged GABA antagonism appeared (Molnár and Nadler, 2000), but this important study was largely ignored by the field for many years. Much to our surprise we discovered subsequently that MNI-Glu did indeed antagonize GABA-A receptors (Ellis-Davies et al., 2007b). It is now widely agreed that all the most widely used caged Glu and GABA probes do show some antagonism toward GABA-A receptors, *especially at concentrations required for 2P uncaging*. We have recently introduced what we think is a general solution to this pervasive problem, namely attaching large neutral or anionic dendrimers to the caged neurotransmitters (Richers et al., 2017). Starting with the strongly antagonistic DEAC450-GABA, we have found that a neutral fifth generation dendrimer reduces the IC-50 90-fold. I would suggest that our cloaking method could provide a general solution to a problem that bedeviled the caged neurotransmitter field since 2000.

### Other Caging Chromophores for 2P Photolysis

The low 2P uncaging cross-section of simple nitroaromatic chromophores has lead chemists to seek to improve the ability of caged Glu to undergo 2P photolysis. The strategy adopted has been to follow rules outlined in 1998 by Marder (Albota et al., 1998) and Reinhardt (Reinhardt et al., 1998), where the size of extended p-electron systems was studied systematically in relation to the 2P absorption cross-section. Goeldner and co-workers pioneered the application of this insight, with several publications. In particular their 2008 description of a large polyaromatic system called BNSF (Gug et al., 2008a), which had a 2P cross-section of 20 GM, was important. Unfortunately the caged glutamate derivative made with the BNSF photochemical protecting group was not very buffer soluble, so no biology was reported with this probe. In contrast, a much smaller aromatic antennae (a methoxybiphenyl) allowed a reasonably soluble caged with a 2P cross-section of 4.5 GM (Gug et al., 2008b). Even with such a promising value compared to MNI-Glu, no 2P biology was reported. We found that a side-by-side biological comparison of a more buffer soluble version of the probe with MNI-Glu reveled that the cuvette properties did not lead to expected improvements in 2P neurobiology, suggesting caution in being able to translate simple chemical measurements to practical physiology (Passlick and Ellis-Davies, 2017). The final chromophore in this series was used to cage GABA in 2012. Electron rich amino-nitrobiphenyls (ANBP) were reported to have 2P cross-section up to 73 GM. But even this exceptional value still required 25 ms irradiation to produce currents of 6 pA from layer 2/3 neurons in brain slices (Donato et al., 2012).

RuBi-Glu also appears to undergo effective 2P uncaging in brain slices (Fino et al., 2009). This probe has been commercially available for many years, but relatively few studies have utilized it (Fino and Yuste, 2011). In contrast, it is RuBi-GABA that has proved popular for visible light (i.e., 1P) uncaging of GABA. Indeed, as noted below, this probe proves a very useful optical partner to MNI-Glu for two-color uncaging using blue and 720 nm 2P light for RuBi and MNI, respectively.

Chemists have reported the development of several other extended p-electron systems for potentially caging neurotransmitters (Korzycka et al., 2015; Cueto Diaz et al., 2016). But like BNSF these all seem to suffer a solubility problem, one which must be inherent to large lipophilic organic molecules. Thus, no neurophysiology has yet been reported using these chromophores.

## Practical Considerations for 2P Uncaging of Glutamate

### Storage

#### MNI-Glu

As noted above, MNI-Glu is exceptionally stable. Solids either as the TFA salt or zwitterion seem indefinitely stable. Solutions at neutral pH can be used after at least 1 year (Amatrudo et al., 2015). Usefully, ACSF solution may be frozen and used subsequently, after filtration, testing for pH and osmolarity. In the case of solutions for local perfusion, Hepes-buffered solutions may be made, frozen in small aliquots, and used very effectively. Solutions of pH > 8 must be avoided as the amide bond can hydrolyze (Kantevari et al., 2016).

#### CDNI and MDNI-Glu

These probes are freely soluble in physiological buffer, solutions of 20 mM are easily made. However, the probes cannot be stored at this pH as they are slightly unstable. There is a similar problem for MDNI-Glu. Thus, solutions must be slightly acidic (ca. pH 4) and frozen for long-term storage. HPLC analysis of solutions frozen for over 2 years at pH 4 show no change (Amatrudo et al., 2015). The probes are easily stored safely as solids for many years. Small aliquots may be made by dissolving in methanol with 0.1% TFA, followed by solvent evaporation on a speedvac.

#### RuBi-Glu

The chemical bond that cages Glu in this probe is not base labile. So solutions of 20 mM can be made and used without any worries over hydrolytic stability. A bigger concern for RuBi-Glu is handling with ambient white light. While MNI-Glu is relatively stable under normal fluorescent lights (these lack much violet light, and have no near-UV), such light readily and rapidly photolyzes RuBi-Glu, so caution must be paid to handling this probe. We use Roscolux filters 13 and 25 to protect MNI and RuBi.

##### Methods of probe application to brain slices

The simplest method is, of course, bath application of a known concentration of probe. However, given that most probes cost >$100 for 10 mg, this can seem prohibitively expensive for many studies. However, it does allow certain types of studied to be performed which other methods do not (Higley and Sabatini, 2008). Notably, if one requires precise pharmacology to be performed across many samples and days, it is the most reliable way to carry out such experiments. A minimum volume for recirculation is about 7 mL, thus care must be taken to monitor solvent evaporation over the period of experimentation.

The simplest alternative to bath application is use of a picospritzer fitted with a normal patch pipette. I recommend Hepes buffered solutions, as opposed to normal ACSF, as this seems the best way to control the pH of the application solution. Very small volumes of solution can last days, leading to substantial cost savings. In the case of any TFA salt, care must be taken to test the pH of the application solution. Here the non-TFA salt of MNI-Glu is quite advantageous, as high concentrations of a zwitterion will not place undue demands on the buffer capacity of solutions that typically only contain 10 mM Hepes. A distinct disadvantage of this method is that local perfusion is indeed quite local, so if one wants to study a large neuron, such as CA1 principal cells the puffing pipette may have to be moved. An elegant alternative has been described by Wang and co-workers who developed a simple “large bore” local perfusion system which we have also used extensively (Civillico et al., 2011). While requiring slightly more material, perhaps 0.2–0.5 mL for a day, this method delivers caged compounds very effectively. And even allows a double barrel application system for side by side comparison of two caged compounds on the same cell (Passlick and Ellis-Davies, 2017).

An elegant method for uncaging power calibration was developed by Sabatini. Concerned about the light scattering nature of brain tissue, they found that one could calibrate the local power dosage at spine head by using the bleaching of Alexa-594 at a known concentration. They found that 40% bleach at any depth below the slice surface would in practice give consistent currents with MNI-Glu uncaging (Bloodgood and Sabatini, 2007). A summary of the chemical properties of important caged glutamate probes is shown in Table 2.

**Table 2:** Photochemical properties of caged glutamate probes.

**Caged Glu**	**ε (**λ**_**max**_)**	**QY**	**ε.QY**	**Time of release (ms)**	**2PuCS (GM/nm)**	**Stability at pH 7.4**	**Commercially available**
CNB	500 (350)	0.14	20	0.021	NR	Slow hydrolysis	Y
MNI	4,500 (336)	0.065	293	NR*	0.06 (740)	Stable	Y
Bhc	17,500 (368)	0.019	329	3–10	1.0 (740)	Stable	N
RuBi	5,600 (450)	0.13	728	0.05	0.14 (800)	Stable	Y
MDNI	6,400 (330)	0.5	3,200	NR*	0.06 (720)	Slow hydrolysis	Y
CDNI	6,400 (330)	0.5	3,200	NR*	0.06 (720)	Slow hydrolysis	N
DEAC450	43,000 (450)	0.39	16,800	NR*	0.5 (900)	Stable frozen	N

*ε, extinction coefficient; λ_max_, absorption maximum; QY, quantum yield; 2PuCS, two-photon uncaging cross section; NR, not reported*.

* *Biology with these p robes implies fast release*.

## Key Experiments Using 2P Uncaging of Glutamate

The first study using 2P uncaging was carried out by Denk in 1994. With this work he established many, but of all, of the key aspects of the new method. Unfortunately his work was essentially limited by the reality that he had to use a probe (CNB-carbamoylcholine) that was originally developed for UV uncaging, and so was not very effective for 2P excitation. Uncaging periods of 30–40 ms were required to produce large ACH receptors currents from cultured cells. In spite of this limitation, Denk revealed that 2P uncaging showed excellent axial resolution, as would be expected for non-linear excitation (Denk, 1994). With the advent of caged glutamate probes designed for 2P neurophysiology, a diverse group of scientists have used the method to study the details of spine and dendritic physiology.

### Optical Quanta

In 2001 Kasai and co-workers established that 2P uncaging of MNI-Glu on cultured neurons enabled diffraction-limited uncaging such that the photo-evoked currents (2pEPSCs) exactly matched individual mEPSCs. Cultured cells allowed staining of pre-synaptic terminals with FM1-43, such that we could visualize pre-synaptic elements which were opposite to AMPA receptor hotspots detected by uncaging, suggesting such receptor clusters were indeed synapses. The evoked currents were found to be correlated with the square of the incident power, imply a true 2P excitation effect (Matsuzaki et al., 2001). Importantly, the high resolution “functional mapping” technique described by Denk was implemented in brain slices with MNI-Glu. We found that APMA receptor currents were strongly correlated with spine head volume. In subsequent experiments it was established that these currents correlated not with a change in conductivity, but with receptor density itself (Tanaka et al., 2005). Furthermore, functional mapping was carried out at several z sections of the brain slice, with the photo-evoked currents showing excellent axial resolution (Figure 3). This set of experiments established for the first time that the very coin of communication in the brain, quantal release of glutamate, could be mimicked optically by neurophysiologists.

### Optical LTP at Visually Designated Synapses

The development of the dual 2P laser microscope by Kasai was the next important advance in this field. Also having two galvanometers, this instrument allowed simultaneous, chromatically independent uncaging, and imaging. The key experiment with this microscope established that an increase in synaptic strength at small spines on CA1 neurons was accompanied by a long-term increase in spine head volume. In other words, LTP had a structural correlate (Matsuzaki et al., 2004). Such optical LTP is probably at a higher frequency (5–10x) than LFS in low Mg^2+^ solutions, yet since glutamatergic input was independent of the pre-synapse, we could select spines of various sizes to show that an increase in volume at individual, isolated was independent from other nearby spines. The photostimulation protocol required ~60 (photochemical) quanta at 1 Hz at 0 mV, or the same input in “zero Mg^2+^” solutions with GFP-labeled neurons that were not patch-clamped. Such experiments established a powerful method for LTP at visually designated spines, and suggested spines were physically isolated biochemical compartments, a reality that many other experiments showed was only partially true.

### 2pEPSCs and 2P Ca^2+^ Imaging

Using the second 2P laser for imaging function rather than structure is a second important application of the dual 2P laser microscope. Given the importance of intracellular Ca^2+^ signaling in neurons, use of low affinity fluorescent Ca^2+^ dyes was the obvious first port of call for this approach. Sabatini and Svoboda both published seminal studies imaging Ca^2+^ in spine heads in 2004 and 2005, respectively (Carter and Sabatini, 2004; Sobczyk et al., 2005). In parallel to these studies, Kasai also reported how the size of the spine neck controlled Ca^2+^ compartmentalization in spines (Noguchi et al., 2005). Sabatini and co-workers published a series of intense studies concerned with the modulation of CaV_2.3_ on spine heads (Ngo-Anh et al., 2005; Bloodgood and Sabatini, 2007; Giessel and Sabatini, 2010). They have found that this voltage-gated ion channel is intimately linked in space to yet another channel on spine heads, the small conductance Ca^2+^-activated potassium (or SK) ion channel. They found that increases in Ca^2+^ concentration via CaV_2.3_ initiated hyperpolarisation, giving rise to apparently conflicting functions of spine Ca^2+^ when compared to NMDA receptors. Sabatini and co-workers concluded that the latter are in a privileged microdomain where large (ca. 10–20 μM), highly local increases in Ca^2+^ via CaV_2.3_ activate adjacent SK channels and the SK channels hyperpolarize spine head membrane potential. The changes in spine head Ca^2+^ from NMDA receptors is only 1–2 μM, so it does not rise to sufficiently high levels to stimulate SK channels. Thus, depending on its origin, spine Ca^2+^ can have bidirectional effects on local potential. All of these studies illustrate the strength of dual 2P microscopy to define the details of the nature of Ca^2+^ signaling in spine heads as the experimenter can immediately link cause and effect at visually designated spine using a stereotypical input.

### Spine LTP Is Local and Non-compartmentalized

The initial single spine studies by Kasai suggested spines were functionally isolated compartments (Matsuzaki et al., 2001). And even though some Ca does spill out of the stimulated spine during 2P uncaging of glutamate, the size and response of each spine in short dendritic segments (ca. 10 mm) are uncorrelated, suggesting spines were isolated biochemcially. However, a seminal study by Harvey and Svoboda revealed that this was an over simplification (Harvey and Svoboda, 2007). They found that spines within 10 mm of a LTP spine seemed to sensitize small, nearby spine to activation to a “sub” optical LTP protocol. Thus, about half the uncaging pulses could be used to induce structural LTP at such spines. In a follow up that appeared shortly after this, the same authors added 2P FRET-FLIM imaging to reveal that *ras* was the biochemical signal transferring input from the initial LTP spine to its neighbors (Harvey et al., 2008). This latter study was carried out in collaboration with Yasuda, who went on to develop and apply a series of FRET-FLIM probes to study in exquisite detail many of the key molecules involved in spine LTP (Lee et al., 2009; Murakoshi et al., 2011, 2017; Zhao et al., 2011; Hedrick et al., 2016; Colgan et al., 2018). Yasuda's work has showed that some molecules remain compartmentalized, whereas other spread to proximate spines with various length and time constants. Crucial to such studies is the continued development of novel FRET-FLIM probes (Zhao et al., 2011; Chu et al., 2016; Laviv et al., 2016). Of course, all of Yasuda's work is built upon the seminal optical LTP study using MNI-Glu by Kasai and co-workers published in 2004 (Matsuzaki et al., 2004).

Complementary to studies on structural LTP have been several reports using 2P uncaging of glutamate to induce structural LTD or synaptogenesis. For example, Kwon and Sabatini showed that 2P uncaging of MNI-Glu on dendritic shafts of L2/3 neurons in brain slices from young mice (P 10–15 days) could induce the growth of new spines. Subsequently the same group showed that neuroligin-1 was crucial for such synaptogenesis (Kwon et al., 2012). Interestingly these spines were always partnered by presynaptic cells, as local electrical stimulation revealed post-synaptic Ca transients in the new spine. Such data are consistent with earlier reports from the Svoboda group that when new spines appeared *in vivo* in adult mice they were found to have a presynaptic bouton with a PSD, which was detected *post-hoc* using EM. In 2012 the Sabatini laboratory used 2P uncaging of MNI-Glu to reveal that synaptogenesis in MSN was activity dependent by chemogenetic control of input onto MSN (Kozorovitskiy et al., 2012). In the same year Zito and co-workers used MNI-Glu to show that *de novo* 2P-induced spinogenesis in slice cultures was dependent on proteasome activity (Hamilton et al., 2012). Zito and co-workers have used 2P uncaging of MNI-Glu to induce LTD at single spines on neurons in slice culture. Starting in 2013, they have shown that “low frequency” 2P stimulation at 0.1 Hz can caused structural LTD. Uncaging power was adjusted to mimic quantal input, and 90 pulses caused spines to shrink in a manner that depended on NMDA, IP_3_, and metabotropic receptors. Shortly after this study appeared, we found that structural LTD was induced by pairing GABA uncaging around spines prior to 2P uncaging of CDNI-Glu in a spike-timing dependent way (Hayama et al., 2013). The latter study was also performed on slice cultures, but 2P input was at 1 Hz. Importantly, the study by Zito demonstrated that LTD was reversible, as MNI-Glu uncaging at 10 Hz not only restored spines to their previous size, but could induce LTP at the LTD spine. Follow up studies by Zito and co-workers showed that the LTD sensitivity could be conferred by local LTP (Oh et al., 2015), and does not always require NMDA receptors (Stein et al., 2015).

### Clustered Spine Signaling Studied Using 2P Uncaging

The non-linear electrical response of neurons is the fundamental of their input-output function. Local non-linearities within branch segments were first reported by Llinas in 1980 (Llinás and Sugimori, 1980). Such dendritic spikes have been studied using local electrode stimulation and uncaging with focused UV lasers (Schiller et al., 2000). In 2006 Magee and co-workers pioneered the use of 2P uncaging to study dendritic spikes with two studies in 2006 (Gasparini and Magee, 2006; Losonczy and Magee, 2006). They established that the local integration of unitary excitatory post-synaptic potentials (uEPSPs^1^) was dependent on the number and distribution of uEPSPs. By patch clamp measurements along main apical dendrite of CA1 neurons the Magee laboratory showed that non-linear output was observed only when the number uEPSPs generated by two-photon uncaging of MNI-Glu at several sites were temporally clustered within a 20-micron dendritic segment. Spatial distribution of the same number of uEPSPs over more than 100 microns produced linear outputs locally (Gasparini and Magee, 2006). In both cases, when the uEPSPs were temporally asynchronous, linear output was recorded locally. Two-photon interrogation of radial oblique dendrites with MNI-Glu revealed that these thin neurites had a different input-output pattern from the main dendritic trunk. In radial oblique dendrites equal non-linear somatic outputs were observed for spatially clustered and distributed multiple uEPSPs (Losonczy and Magee, 2006). This study also provided an estimate of the number of uEPSPs required on average for dendritic spike by showing that two-photon uncaging at ~20–25 spines (with a somatic potential of 0.25 mV per spine) could evoke a dendritic spike. Thus, 6–7 2pEPSPs of 0.75 mV can have approximately the same somatic output as 25 uEPSPs of 0.25 mV. Finally, the Magee laboratory established that if clustered inputs were positioned close the main trunk (ca. 20 microns), a significantly larger synaptic input was required to induce a dendritic spike when compared to that required for inputs positioned near the end (>90 microns from the trunk) of a dendrite. This is because the main trunk acts as a current sink for proximal inputs, whereas because the input resistance increases toward the terminus of the dendrite this prevents current loss and reduces the amount of synaptic input required for a dendritic spike. Following this, Hausser et al. used such patterned inputs to verify predictions from cable theory by Wilfred Rall, confirming that the direction of local dendritic input (toward the cell was stronger than away from it) conditioned the strength of the Ca^2+^ signal (Branco et al., 2010).

In 2008 the Magee laboratory explored the effects of clustered inputs on the *strength of the output*, and discovered that some branches were plastic. Significant differences between the non-linear electrical responses of individual dendrites were discovered. Stereotypical two-photon uncaging of MNI-Glu at spatially clustered groups of spine heads on almost 500 basal and proximal radial oblique dendrites of CA1 neurons revealed that non-linear responses could be categorized bimodally into “strong” and “weak” groups with a 10-fold difference in rate of change of potential (Losonczy et al., 2008). Interestingly they found that weak branches could be converted into strong ones by bath application of carbachol and local theta stimulation for about 20 min, and called this new form of synaptic plasticity “branch-strength potentiation.” In Kv1.4 knockout mice such changes were not observed. Following this study, Tonegawa and coworkers used clustered multi-spine uncaging of MNI-Glu to determine if changes branch-strength potentiation were accompanied by local protein translation (Govindarajan et al., 2011). They found that when 10–20 spines were synchronously stimulated in the presence of forskolin a few spines (average 4) within this group underwent structural LTP that lasted for at least 240 min (sL-LTP). If forskolin was not present the initial volume change was not sustained beyond 150 min. Note, previous studies (Matsuzaki et al., 2004; Harvey and Svoboda, 2007; Harvey et al., 2008; Patterson et al., 2010) did not normally go beyond 100 min so did not detect this slow volume loss. There was also a small group of spines (average 1–2) from the initial group of unpotentiated spines that exhibited sL-LTP from a second round of clustered uncaging, but without forskolin present. This “local priming” is similar to that reported by Svoboda and coworkers for single spine structural LTP. Yasuda and co-workers have shown that induction of structural LTP at a few (3–7) spatial dispersed spines can initiate ERK translocation to the nucleus of CA1 neurons where the protein upregulated transcription factors (Zhai et al., 2013). This fascinating study revealed that a few spines on the same branch did not have this effect, whereas 7 spines distributed over as much as 200 microns could produce an integrated nuclear increase in ERK. Interestingly the “timing window” for these effects had to be >40 min. These recent studies by the Tonegawa and Yasuda laboratories suggest that CA1 pyramidal neurons can encode both clustered and distributed synaptic inputs to control protein translation and transcription in distinctly different ways.

These 2P studies were conducted on pyramidal cells in the cortex. What about neurons in other brain regions? Surmeier and co-workers have used multi-spine head uncaging of MNI-Glu on medium spiny neurons (MSN) to show that sustained upstates can be generated by targeting clustered groups of 10 spines more than 100 microns from the soma (Plotkin et al., 2011). Stimulation of clusters at a distance of 40–60 microns reduced the length of the upstate from 120 to 30 ms. T-type Ca channels and NMDA receptors were found to be responsible for these upstates, but other voltage gated dendritic ion channels had no role. Interestingly, direct and indirect pathway MSN had similar non-linear properties.

### Two-Color Actuation: 2P Uncaging of Glutamate Paired With Blue-Sensitive Probes

Most of the studies using using 2P uncaging of glutamate used our original protocol with Ti:sapphire lasers mode-locked at about 720 nm to photolyze MNI-Glu. Since the absorbance of visible light above about 420 nm by MNI is low, and is zero in the blue region, this provides a chromatic channel to allow two-color actuation. Several studies have appeared using this approach. The first example of this approach was a very elegant study by Oertner and co-workers in 2008 (Zhang et al., 2008). This work took advantage of the poor response of ChR2 toward 2P excitation at 720 nm, thus expressing this light-gated ion channel in neurons allowed a 2-color induction of LTP at single spines. Blue light was used to initiate an action potential, with 2P uncaging of MNI-Glu timed to coincide with this in a form of spike-timing dependent plasticity (STDP). In the same year Kasai and co-workers presented a detailed study of the role of protein synthesis in STDP, but we used whole-cell patch-clamp in a more Classical approach (Tanaka et al., 2008). The use of blue light to induce spikes by Oertner circumvented the patch-clamp requirement, and in so doing allowed them to use FRET-FLIM imaging of CaMKII to quantify the time course of kinase action during plasticity at single spines.

More recently, Kasai and co-workers used the poor response of RuBi-GABA toward 2P activation to study effects of local inhibition of spine LTD and LTP (Hayama et al., 2013). Pairing blue photolysis of this probe with CDNI-Glu (uncaging at 720 nm), they concluded that local inhibition could condition the response of single spines to a STDP-like LTD protocol (but not LTP). Further, the LTD effect was not entirely local, as with about 15 microns induced by the spread of activated cofilin. Independent studies by Higley and co-workers revealed that using the same chemical probes, discovered that a subset of spines on L2/3 neurons in the PFC receive both glutamatergic and GABAergic synaptic inputs (Chiu et al., 2013). On disynaptic spines, inhibition was sufficient to sculpt spine Ca^2+^ transients, modeling suggested that the resistance of the spine neck was sufficient to isolate such spines from any local dendritic inhibition. Finally, the development of the blue-light responsive DEAC450 (Olson et al., 2013a; Agarwal et al., 2017) will enable the study of the symbiotic nature of intracellular signaling and excitatory input.

## Summary

Two-photon uncaging of glutamate is a well-established technique. It has been widely used to probe the electrical and biochemical properties of individual spines. Its spatial precision provides a powerful means of optical interrogation of these privileged domains. Further, the ability to address the physiological synergism amongst spines can be studied in a unique way by multi-spine stimulation using 2P uncaging. Chromatically complementary chromophores provide further opportunities for physiologists to probe parallel signaling pathways using 2-color photoactuation. Many genetically-encoded probes do not normally respond effectively to the short 2P wavelengths used for glutamate uncaging, thus “old” and “modern” optical methods could work together in an uniquely powerful way for many future experiments.

## Author Contributions

The author confirms being the sole contributor of this work and has approved it for publication.

### Conflict of Interest Statement

The author declares that the research was conducted in the absence of any commercial or financial relationships that could be construed as a potential conflict of interest.

## Footnotes

^1^Note this standard notation for quantal signals is sometimes used for “uncaging EPSPs”. I find such use in this context confusing, as photochemical “uEPSPs” are used to mimic quantal uEPSPs. For this reason we chose, in 2001, “2pEPSC” to denote the currents evoked by two-photon uncaging of glutamate. Laser energy can be tuned to evoke a postsynaptic signal that mimics either an uEPSC or uEPSP, or such signals can be made supraquantal by increasing the uncaging energy.
